# Ventricular Septal Rupture Despite Early Pharmaco-Invasive Reperfusion in Anterior STEMI With Favorable Surgical Outcome

**DOI:** 10.7759/cureus.107945

**Published:** 2026-04-29

**Authors:** Jonhatan M Cota-Arce, Adan Becerril Ponce, Luis A Morales-Díaz, Luis M Alvárez Sánchez, Claudia G Cota-Arce

**Affiliations:** 1 Cardiology, Instituto Mexicano del Seguro Social, Mexico City, MEX; 2 Cardiothoracic Surgery, Instituto Mexicano del Seguro Social, Mexico City, MEX; 3 General Medicine, Universidad de Sonora, Hermosillo, MEX

**Keywords:** anterior st-elevation myocardial infarction, mechanical complications of myocardial infarction, post-infarction ventricular septal defect, surgical repair, ventricular septal rupture

## Abstract

Ventricular septal rupture (VSR) is a rare but catastrophic mechanical complication of acute myocardial infarction, even in the contemporary reperfusion era. Although early reperfusion strategies have significantly reduced its incidence, they do not completely eliminate the risk. We report the case of a 76-year-old man with a history of hypertension and no prior structural heart disease who presented with an anterior ST-elevation myocardial infarction (STEMI). He received tenecteplase 2 hours and 37 minutes after symptom onset and was subsequently referred for routine percutaneous coronary intervention (PCI), during which a stent was implanted in the left anterior descending (LAD) artery.

During in-hospital monitoring, a new harsh holosystolic murmur was detected. Transthoracic echocardiography revealed an 11-mm interventricular septal defect with left-to-right shunting, which was confirmed by color and spectral Doppler. Despite this mechanical complication, the patient remained hemodynamically stable and did not require vasopressor support. He underwent successful surgical repair using an infarct exclusion technique with a bovine pericardial patch. The postoperative course was favorable, with no need for mechanical circulatory support, extubation within the first 12 hours, no requirement for high-dose vasoactive amines, and no residual shunt on follow-up echocardiography. He was discharged home with outpatient follow-up.

This case highlights that post-infarction VSR may occur despite timely pharmaco-invasive reperfusion, particularly in anterior STEMI, and underscores the importance of continued clinical vigilance even after apparently successful reperfusion. Early recognition through physical examination and echocardiography, together with prompt multidisciplinary management, may allow for favorable outcomes in selected patients.

## Introduction

Ventricular septal rupture (VSR) is a rare but catastrophic mechanical complication of acute myocardial infarction (AMI). Although its incidence has markedly declined in the reperfusion era, it remains associated with substantial morbidity and mortality. Contemporary data suggest that post-infarction VSR occurs in approximately 0.21% of patients with ST-segment elevation myocardial infarction (STEMI), making it an uncommon but highly lethal event in modern practice [1,2]. VSR is more frequently observed after transmural infarction and is particularly associated with anterior STEMI due to left anterior descending (LAD) artery occlusion [1,3]. Anterior infarction is clinically important because it often involves a large ischemic territory and extensive septal necrosis, increasing the likelihood of structural myocardial failure. Reported risk factors for post-infarction VSR include advanced age, female sex, hypertension, a first myocardial infarction, anterior infarction, absence of prior smoking, poor collateral circulation, and delayed or ineffective reperfusion [1,2].

This complication usually develops within the first week after infarction, most commonly between days 3 and 5, although earlier presentation may also occur [1,4]. Current European guidance describes ventricular septal rupture as a mechanical complication that typically appears 3-7 days after AMI, which remains a useful clinical window for surveillance [4]. Clinically, the appearance of a new harsh holosystolic murmur, rapid hemodynamic deterioration, pulmonary congestion, or signs of cardiogenic shock should immediately raise suspicion for VSR [2,4].

The prognosis of post-infarction VSR remains poor, particularly in the absence of definitive closure. Historically, medical therapy alone has been associated with near-universal mortality, whereas surgical repair offers the only realistic chance of survival in most patients [1-3]. Even with surgery, early mortality remains high. In a large analysis from The Society of Thoracic Surgeons database, operative mortality was 42.9% overall, reaching 54.1% when repair was performed within seven days of myocardial infarction, compared with 18.4% when surgery was delayed beyond seven days; however, this difference is strongly influenced by survivor bias, since the sickest patients often require urgent intervention [5].

Diagnosis relies primarily on transthoracic echocardiography with Doppler, which confirms the septal defect, identifies the direction of shunting, and assesses ventricular function and associated complications [2]. Management requires rapid hemodynamic assessment and a multidisciplinary approach. Initial treatment may include vasodilators, diuretics, vasoactive agents, and, in unstable patients, mechanical circulatory support such as an intra-aortic balloon pump or extracorporeal support as a bridge to closure [1,2]. Surgical repair remains the standard definitive treatment for most patients, whereas transcatheter closure may be considered in selected cases, particularly in patients at prohibitive surgical risk, as a bridge strategy, or in residual postoperative shunts [1,2].

In this context, we present the case of an elderly patient with anterior STEMI who developed post-infarction ventricular septal rupture despite timely fibrinolysis with tenecteplase followed by successful LAD percutaneous coronary intervention. Notably, the patient remained hemodynamically stable despite the presence of VSR, which allowed for delayed elective surgical repair without the need for mechanical circulatory support and resulted in a favorable postoperative outcome. This case highlights that early reperfusion reduces, but does not eliminate, the risk of mechanical complications and underscores the importance of continued clinical vigilance even after apparently successful reperfusion, particularly in patients with atypical and stable presentations [1,2].

## Case presentation

**Figure 1:**
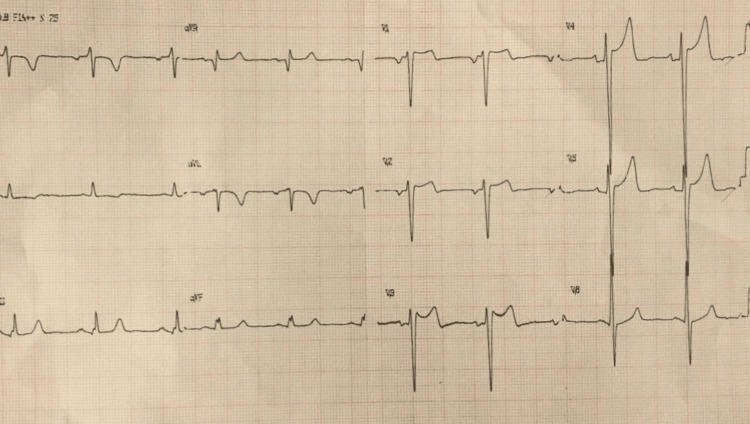
Initial electrocardiogram on admission Initial electrocardiogram showing ST-segment elevation in the anterior precordial leads (V2–V4), consistent with acute anteroseptal ST-elevation myocardial infarction.

A 76-year-old man with a history of hypertension and no known prior structural heart disease developed acute chest pain while performing exertional activities at home around midday. He described oppressive chest pain radiating to both arms, associated with nausea and diaphoresis. He presented to the hospital approximately two hours after symptom onset, where an electrocardiogram demonstrated ST-segment elevation in the anteroseptal leads, consistent with anterior ST-elevation myocardial infarction (STEMI) (Figure 1). Given the absence of on-site catheterization laboratory facilities for diagnostic coronary angiography and percutaneous coronary intervention, fibrinolytic therapy with tenecteplase was administered 2 hours and 37 minutes after symptom onset, with subsequent improvement in symptoms and indirect signs of reperfusion (>50% ST-segment resolution and relief of chest pain). The patient was then referred to a tertiary care center with a catheterization laboratory available. On arrival, he was hemodynamically stable and asymptomatic, and a pharmaco-invasive strategy was pursued.

**Figure 2:**
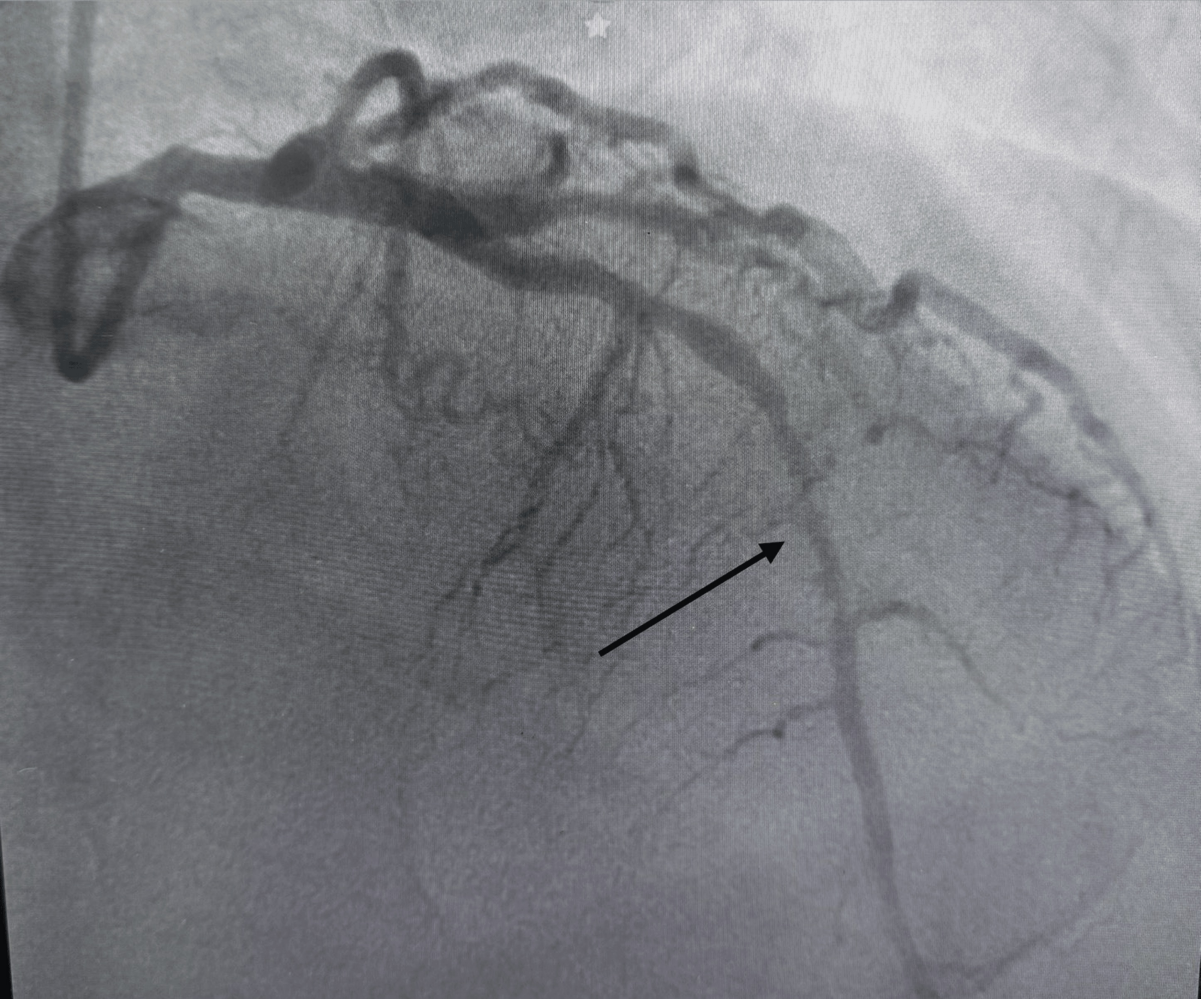
Diagnostic coronary angiography before percutaneous coronary intervention Diagnostic coronary angiography performed before percutaneous coronary intervention showing partial reperfusion in the left anterior descending artery, with Thrombolysis in Myocardial Infarction (TIMI) 2 flow at the time of catheterization. A significant lesion in the mid-left anterior descending artery is still observed (arrow).

Coronary angiography revealed single-vessel coronary artery disease with a significant mid-LAD lesion (75% stenosis, diffuse type B1 according to American Heart Association/ American College of Cardiology (AHA/ACC) classification) and Thrombolysis in Myocardial Infarction (TIMI) 2 flow prior to intervention (Figure 2), consistent with incomplete reperfusion following fibrinolysis. Percutaneous coronary intervention (PCI) with drug-eluting stent implantation in the mid and distal LAD segments was successfully performed approximately six hours after symptom onset, achieving final TIMI 3 flow. The patient was subsequently admitted to the coronary care unit, hemodynamically stable and without chest pain. No complications related to cardiac catheterization were observed, and there was no need for inotropic or vasopressor support. During in-hospital monitoring, 36 hours after admission to the coronary care unit, a new harsh holosystolic murmur best heard at the mesocardium was detected, consistent with an early ventricular septal rupture, raising suspicion for a mechanical complication.

On admission to the coronary care unit, the patient had a blood pressure of 110/70 mmHg and a heart rate of 76 beats per minute. He required low-flow oxygen via nasal cannula at 2 L/min, maintaining oxygen saturation above 94%. Arterial blood gas analysis showed a pH of 7.44, pCO₂ of 35 mmHg, pO₂ of 73 mmHg, lactate of 1.2 mmol/L, and bicarbonate of 24.6 mmol/L. Laboratory values included a creatinine of 0.54 mg/dL, urea of 34 mg/dL, and BUN of 16 mg/dL. These parameters remained stable and within similar ranges throughout hospitalization up to the time of surgical intervention.

**Figure 3:**
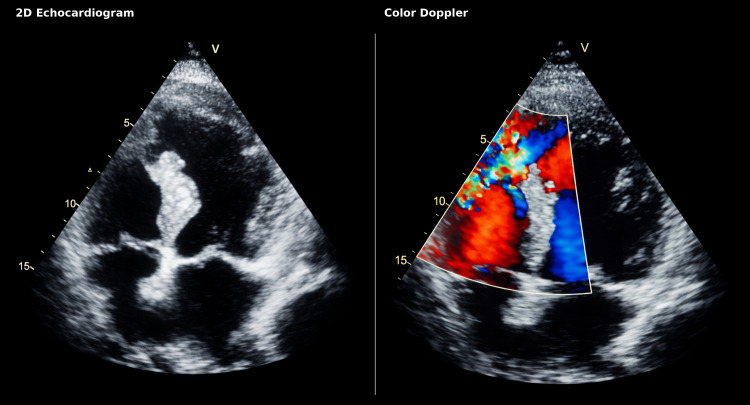
Apical four-chamber echocardiographic view The images demonstrate inferoseptal wall thinning and post-infarction ventricular septal rupture. Apical four-chamber transthoracic echocardiographic view showing thinning of the apical inferoseptal septum with an irregular “bite-like” appearance secondary to septal rupture on two-dimensional imaging (left panel). Color Doppler (right panel) demonstrates a left-to-right shunt across the interventricular septum, confirming post-infarction ventricular septal rupture.

A transthoracic echocardiogram was obtained and demonstrated an 11-mm ventricular septal defect located in the apical muscular interventricular septum, consistent with a simple ventricular septal rupture, with left-to-right shunting Qp:Qs 2.3 (Figure 3). The diagnosis of post-infarction ventricular septal rupture (VSR) was confirmed by color and spectral Doppler. Left ventricular systolic function was preserved, with an ejection fraction of 61%. Right ventricular function was normal, with a tricuspid annular plane systolic excursion (TAPSE) of 24 mm and tricuspid annular systolic velocity of 18 cm/s. Estimated pulmonary capillary wedge pressure was 21 mmHg, with a cardiac output of 5.8 L/min and a cardiac index of 2.9 L/min/m². No papillary muscle rupture was identified, and only mild mitral regurgitation was present, with a vena contracta of 3 mm. Despite these findings, the patient remained hemodynamically stable, with no evidence of cardiogenic shock and no requirement for vasopressor support.

**Figure 4:**
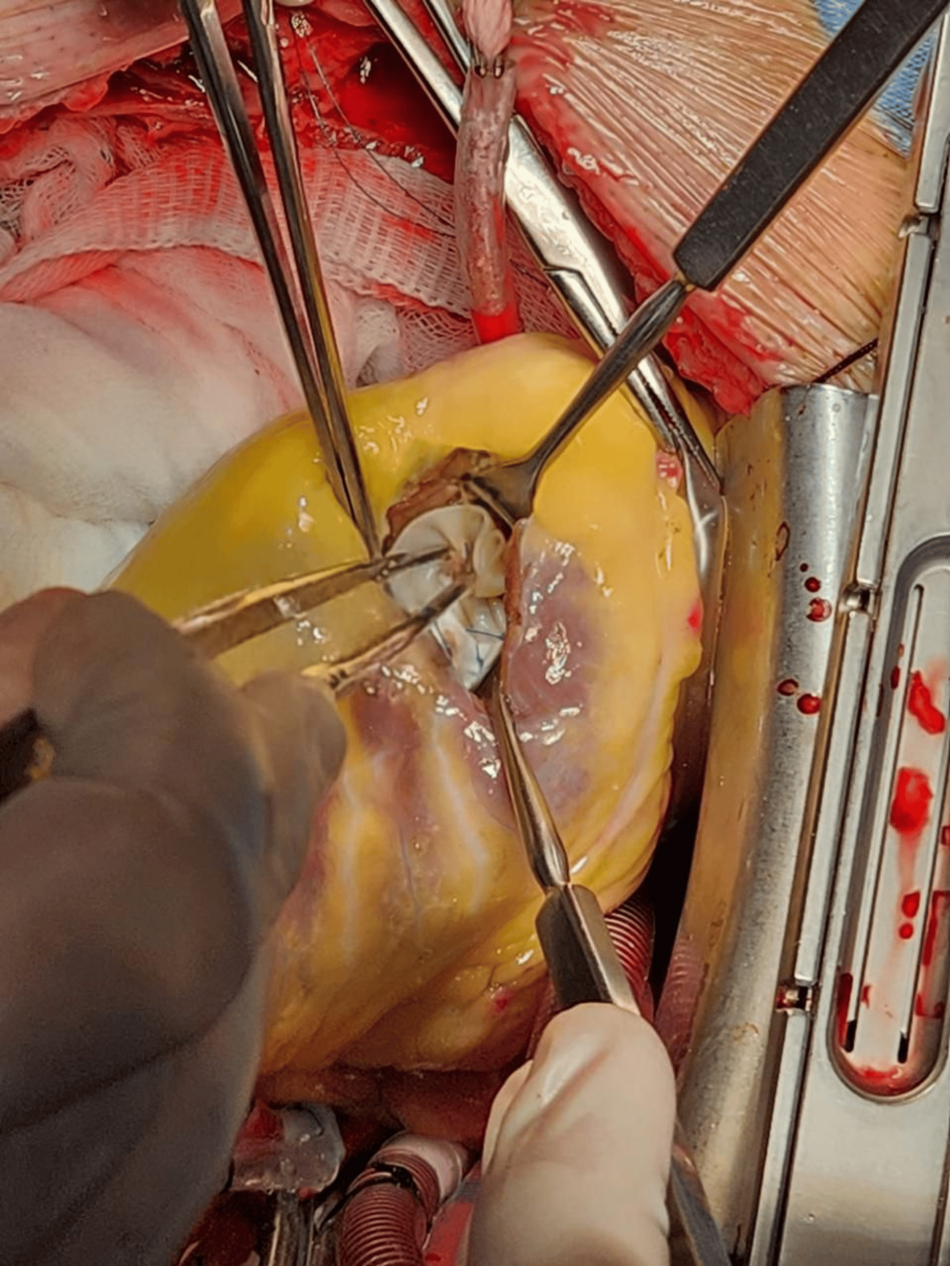
Intraoperative repair of post-infarction ventricular septal rupture Intraoperative view showing surgical repair of the post-infarction ventricular septal rupture. A bovine pericardial patch is being positioned to close the septal defect through the left ventriculotomy.

The case was discussed in a multidisciplinary medical-surgical session, and elective surgical repair was planned after 11 days of clinical stability in the coronary care unit. The patient underwent repair using an infarct exclusion technique. A left ventriculotomy was created through the infarcted apical myocardium and extended approximately 10-15 mm away from the left anterior descending (LAD) artery. Intraoperative findings revealed a funnel-shaped ventricular septal defect located approximately 15 mm from the LAD artery. A bovine pericardial patch was sutured to the endocardium of the non-infarcted interventricular septum and the anterior ventricular wall, thereby excluding the infarcted septal segment from the left ventricular cavity (Figure 4). 

**Figure 5:**
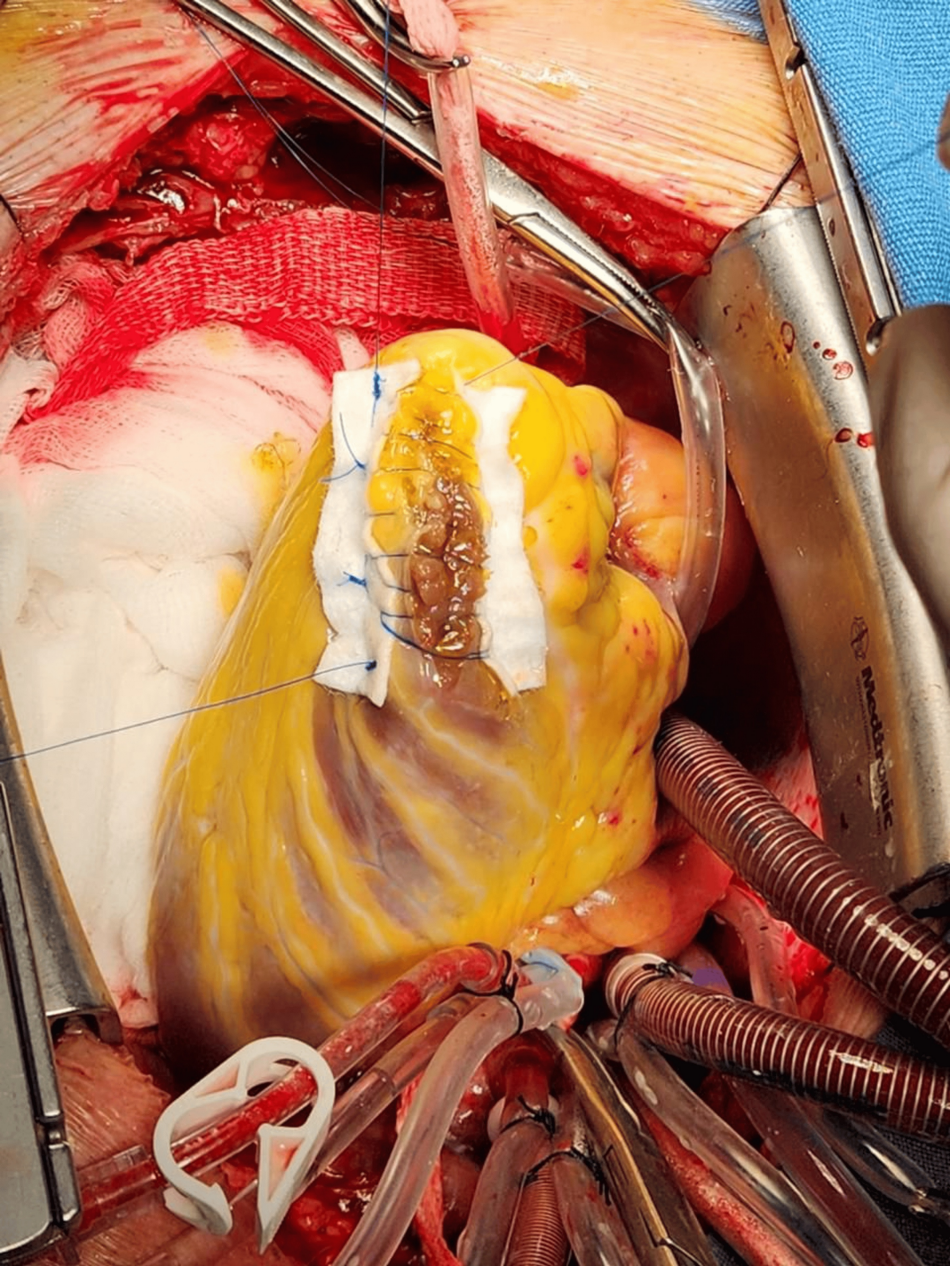
Closure of the left ventriculotomy using the sandwich technique Intraoperative view showing closure of the left ventriculotomy after repair of the post-infarction ventricular septal rupture with a bovine pericardial patch. The ventriculotomy was reinforced using a sandwich technique.

Once the patch had been positioned and secured, the left ventriculotomy was closed using a reinforced sandwich-like technique, with buttressing of the ventriculotomy edges to reduce tension on the friable infarcted myocardium (Figure 5). Total intraoperative blood loss reported was 600 mL, with a cardiopulmonary bypass time of 2 hours and 15 minutes and an aortic cross-clamp time of 1 hour and 10 minutes.

The procedure was completed without intraoperative complications. In the immediate postoperative period, the patient did not require mechanical circulatory support and was extubated within the first 12 hours after surgery. He never required high-dose vasoactive amines or inotropic support. Follow-up transthoracic echocardiography demonstrated a left ventricular ejection fraction of 48% and successful closure of the distal interventricular septum with a patch, with no evidence of residual shunting. The patient had a favorable recovery and was ultimately discharged home 28 days after symptom onset, with outpatient follow-up.

## Discussion

Post-infarction ventricular septal rupture (VSR) remains one of the most devastating mechanical complications of acute myocardial infarction. Although its incidence has markedly declined in the reperfusion era, it is still associated with substantial early mortality, particularly in patients who develop heart failure or cardiogenic shock [1-3]. Contemporary data suggest that VSR occurs in approximately 0.2% of patients with ST-segment elevation myocardial infarction (STEMI), making it uncommon in modern practice but still clinically significant because of its catastrophic hemodynamic consequences [1,2,6].

Our case is notable because ventricular septal rupture (VSR) developed despite a pharmaco-invasive reperfusion strategy initiated within guideline-recommended timeframes, including fibrinolysis with tenecteplase administered 2 hours and 37 minutes after symptom onset, followed by PCI of the left anterior descending (LAD) artery approximately 6 hours after symptom onset. Initial fibrinolysis was associated with indirect signs of reperfusion (>50% ST-segment resolution and relief of chest pain), although coronary angiography demonstrated incomplete reperfusion with TIMI 2 flow prior to PCI. VSR was identified within 36-48 hours after reperfusion, consistent with an early mechanical complication. This finding reinforces an important clinical concept: although early reperfusion substantially reduces the incidence of mechanical complications, it does not completely eliminate the risk [1-3]. In anterior STEMI, particularly when the LAD territory is involved, the extent of myocardial necrosis and septal damage may still be sufficient to result in structural rupture, even after restoration of epicardial flow [1,3].

Several predictors of post-infarction VSR have been described, including advanced age, female sex, hypertension, first myocardial infarction, anterior infarction, absence of prior smoking, poor collateral circulation, and delayed or ineffective reperfusion [1,2,7]. Although our patient was male, he had several recognized risk features, including advanced age, hypertension, first myocardial infarction, and anterior STEMI involving the LAD territory. These factors may have contributed to the development of septal rupture despite reperfusion therapy [1,2].

The timing of VSR is also clinically important. It typically occurs within the first week after infarction, most commonly between days 3 and 5, although earlier presentation may occur [1-4,7]. This temporal pattern is consistent with the progression of coagulative necrosis and enzymatic digestion of infarcted myocardium, which progressively weakens the septal tissue [1,3]. In this context, our case underscores the need for continued surveillance even after apparent reperfusion success. The detection of a new harsh holosystolic murmur prompted immediate echocardiographic assessment and allowed diagnosis before hemodynamic collapse occurred.

A particularly relevant aspect of this case is the favorable postoperative evolution. Post-infarction VSR is often accompanied by rapid clinical deterioration, vasopressor dependence, need for mechanical circulatory support, and high operative mortality [1,2,5]. In contrast, our patient remained hemodynamically stable, did not require preoperative vasopressor support, underwent planned surgical correction, and had an uncomplicated postoperative course with early extubation, no need for mechanical circulatory support, no requirement for high-dose vasoactive amines, and no residual shunt on follow-up echocardiography. This unusually favorable outcome likely reflects early recognition of the complication before the onset of shock, prompt multidisciplinary evaluation, and successful definitive repair [1,2].

The management of post-infarction VSR remains challenging. Transthoracic echocardiography with Doppler is the diagnostic cornerstone because it confirms the defect, characterizes the direction of shunting, and evaluates ventricular function and associated complications [2]. Surgical repair remains the standard definitive therapy for most patients, whereas transcatheter closure may be considered in selected cases, such as those with prohibitive surgical risk, residual postoperative shunts, or as a bridge strategy [1,2]. In our patient, surgical correction was performed using an infarct exclusion technique with a bovine pericardial patch, followed by reinforced closure of the left ventriculotomy. This approach achieved complete closure of the defect without residual shunting and was associated with excellent short-term recovery.

The issue of surgical timing deserves mention. Data from The Society of Thoracic Surgeons database showed an overall operative mortality of 42.9%, with higher mortality among patients operated on within 7 days of myocardial infarction than among those repaired later; however, this difference is strongly influenced by survivor bias because more unstable patients require urgent surgery [5]. Therefore, the timing of intervention should be individualized according to hemodynamic status, anatomy of the defect, tissue friability, and institutional expertise [1,2,5]. Our patient’s stability allowed planned operative management, which may have contributed to the favorable result.

Overall, this case illustrates two clinically important points. First, ventricular septal rupture may still occur despite timely fibrinolysis and subsequent PCI in anterior STEMI. Second, early clinical suspicion, prompt echocardiographic confirmation, and timely surgical repair in a hemodynamically stable patient may result in an outcome that is considerably better than expected for this otherwise highly lethal complication [1-3].

## Conclusions

Ventricular septal rupture remains a rare but life-threatening mechanical complication of acute myocardial infarction, even in the era of timely reperfusion. This case illustrates that ventricular septal rupture may still occur despite early fibrinolysis followed by percutaneous coronary intervention in anterior STEMI. It also highlights the importance of continued clinical vigilance after apparently successful reperfusion, as the detection of a new murmur allowed prompt echocardiographic diagnosis before hemodynamic deterioration. Early multidisciplinary evaluation and timely surgical repair may lead to favorable outcomes in selected hemodynamically stable patients. However, given that this is a single case with short-term follow-up, the long-term durability of the repair and the generalizability of these findings cannot be established.
